# Identification of cutoff points for Homeostatic Model Assessment for Insulin Resistance index in adolescents: systematic review

**DOI:** 10.1016/j.rppede.2016.01.004

**Published:** 2016

**Authors:** Maria Izabel Siqueira de Andrade, Juliana Souza Oliveira, Vanessa Sá Leal, Niedja Maria Silva da Lima, Emília Chagas Costa, Nathalia Barbosa de Aquino, Pedro Israel Cabral de Lira

**Affiliations:** Universidade Federal de Pernambuco (UFPE), Recife, PE, Brazil

**Keywords:** Insulin resistance, Adolescent, ROC curve, Review

## Abstract

**Objective:**

To identify cutoff points of the Homeostatic Model Assessment for Insulin Resistance (HOMA-IR) index established for adolescents and discuss their applicability for the diagnosis of insulin resistance in Brazilian adolescents.

**Data source:**

A systematic review was performed in the PubMed, Lilacs and SciELO databases, using the following descriptors: "adolescents", "insulin resistance" and "Receiver Operating Characteristics Curve". Original articles carried out with adolescents published between 2005 and 2015 in Portuguese, English or Spanish languages, which included the statistical analysis using Receiver Operating Characteristics Curve to determine the index cutoff (HOMA-IR) were included.

**Data synthesis:**

A total of 184 articles were identified and after the study phases were applied, seven articles were selected for the review. All selected studies established their cutoffs using a Receiver Operating Characteristics Curve, with the lowest observed cutoff of 1.65 for girls and 1.95 for boys and the highest of 3.82 for girls and 5.22 for boys. Of the studies analyzed, one proposed external validity, recommending the use of the HOMA-IR cutoff>2.5 for both genders.

**Conclusions:**

The HOMA-IR index constitutes a reliable method for the detection of insulin resistance in adolescents, as long as it uses cutoffs that are more adequate for the reality of the study population, allowing early diagnosis of insulin resistance and enabling multidisciplinary interventions aiming at health promotion of this population.

## Resumo

**Objetivo:**

Identificar os pontos de corte do índice Homeostatic Model Assessment for Insulin Resistance (HOMA-IR) estabelecidos para adolescentes e discutir a sua aplicabilidade para o diagnóstico da resistência à insulina em adolescentes brasileiros.

**Fontes de dados:**

Revisão sistemática feita nas bases de dados PubMed, Lilacs e SciELO com os descritores "Adolescentes", "Resistência à insulina" e "Curva ROC". Foram incluídos artigos originais, publicados entre 2005 e 2015, conduzidos com adolescentes, no idioma português, inglês ou espanhol e incluindo análise estatística com uso da curva ROC para determinação dos pontos de corte do índice (HOMA-IR).

**Síntese dos dados:**

Foram identificados 184 artigos e, após a aplicação das etapas do procedimento, foram selecionados sete para compor a revisão. Todos os estudos selecionados estabeleceram seus pontos de corte com a curva ROC. O menor ponto de corte observado foi de 1,65 para meninas e 1,95 para meninos e o maior de 3,82 para meninas e 5,22 para meninos. Dos estudos analisados, um propôs validade externa, recomendando o uso do ponto de corte do HOMA-IR >2,5 para ambos os sexos.

**Conclusões:**

O índice HOMA-IR constitui-se em método confiável para deteção da resistência insulínica em adolescentes, desde que usados os pontos de corte que mais se adequem à realidade da população em estudo, o que permite um diagnóstico precoce da resistência à insulina e possibilita intervenções multiprofissionais para a promoção da saúde dessa população.

## Introduction

Adolescence is a critical period for the onset of obesity and other metabolic disorders associated with body fat accumulation. Adolescents with excess weight have a high risk of becoming obese adults and are prone to developing cardiovascular diseases.^1^^,^ 2

Excessive accumulation of body fat, particularly fat located in the central or visceral region, favors the increase in free fatty acids in the bloodstream, which may impair insulin signaling, decreasing the sensitivity of receptors on cell membranes and resulting in insulin resistance (IR).^3^

Brazilian studies have detected the prevalence of IR in the age range of adolescence and have reported prevalence rates ranging from 6.5% to 90.8% in adolescents with and without excess weight.^3^^-^ 5 The most commonly used methods for determining IR in epidemiological studies are obtained from practical formulas that use fasting glucose and insulin levels, as the Fasting Glucose/Insulin Ratio (FGIR), the Quantitative insulin sensitivity check index (QUICKI) and the Homeostatic Model Assessment for Insulin Resistance (HOMA-IR), which has been frequently validated in children and adolescents and is recommended as the most sensitive and specific method for assessing insulin sensitivity in this population.^6^^-^ 8 It is noteworthy that one of the important aspects to be observed in the successful application of HOMA-IR index in a given population is the use of specific cutoffs for gender, ethnicity, age and/or sexual maturation level (if used in adolescents). For this reason, several cutoff points have been recommended for the diagnosis of IR based on the index.^9^^-^ 12 The objective of this study was to identify HOMA-IR index cutoffs established for adolescents and discuss their applicability for the diagnosis of IR in Brazilian adolescents.

## Method

### Literature search strategy

A systematic literature review of scientific articles on the topic "Insulin resistance in adolescents" was carried out, taking into account the following guiding question: "what are the cutoffs for HOMA-IR index established for IR determination in adolescents with and without metabolic syndrome in observational studies?".

The definition of the research question was structured according to the acronym PECO, recommended by the Methodological Guidelines for the preparation of systematic review and meta-analysis of comparative observational studies on risk factors and prognosis, in which each letter corresponds to a component of the guiding question: P - population, E - exposure, C - Control, O - Outcome.^13^ After determining the question, a search was carried out in the PubMed, Lilacs and SciELO databases.

To search used the following descriptors: "adolescent", "Insulin resistance" and "ROC (Receiver Operating Characteristic) curve". The terms present in the model were found in the list of Medical Subject Headings (Mesh), available from the US National Library of Medicine, and the list of Health Sciences Descriptors, available on the BVS portal.

The search in PubMed used the following strategy: ("adolescent" [Mesh Terms] OR "adolescent" [All Fields] OR "adolescents" [All Fields]) AND ("insulin resistance" [Mesh Terms] OR ("insulin" [All Fields] AND "resistance" [All Fields]) OR "insulin resistance" [All Fields]) AND ("roc curve" [MeSH Terms] OR ("roc" [All Fields] AND "curve" [All Fields]) OR "roc curve" [All Fields]). In the Lilacs and SciELO databases, the search was carried out using the expression: (tw:[adolescentes]) AND (tw:[resistência à insulina]) AND (tw:[curva roc]) AND (instance: regional).

The methodological procedure used to carry out this research was complete and finalized on March 1st, 2015.

### Study selection

The articles identified during the database search were selected after the reading of the titles, followed by the abstracts and full texts, when indicated. The procedure was independently carried out by two researchers, taking into account the predefined inclusion criteria: original article, published in the last 10 years (between 2005 and the search end date), carried out with Adolescents, written in Portuguese, English or Spanish, including statistical analysis using Receiver Operating Characteristics Curve to determine the cutoff points for HOMA-IR index.

After article selection, the Kappa index was applied^14^ to analyze the agreement between the two researchers and an excellent/almost perfect agreement was found (*κ*=0.90). In case of disagreement, the studies were discussed in a meeting with the authors for evaluation and consensus on their inclusion in this review.

The entire description procedure for identification and selection of studies was based on the guideline Preferred Reporting Items for Systematic Reviews (Prisma).^15^

### Analysis of article quality

Article quality was assessed according to the initiative of Strengthening the Reporting of Observational Studies in Epidemiology (Strobe), translated into Portuguese.^16^ The checklist comprising Strobe includes 22 questions divided into six groups: Title and Abstract, Introduction, Methods, Results, Discussion and Other Information. Thus, the articles included in this review were scored from 0 to 22, which were later transformed into percentages for better qualitative analysis.

Considering the nature of the initial search for observational studies, of the eligibility criteria to conduct the findings to directed articles and the small number of studies in Brazil, it was decided to include all eligible articles, regardless of the achieved score.

### Data extraction

Data extraction was performed using Microsoft Excel program, version 2007 using a protocol created by the researchers, in which the following data were included: article title, author, place and year of publication, sample size, characteristics of the study population, age range, cutoff based on the HOMA-IR index, sensitivity and specificity of the cutoff determined through the HOMA-IR index, limitations and external validation of the selected studies.

## Results

**Figure 1:**
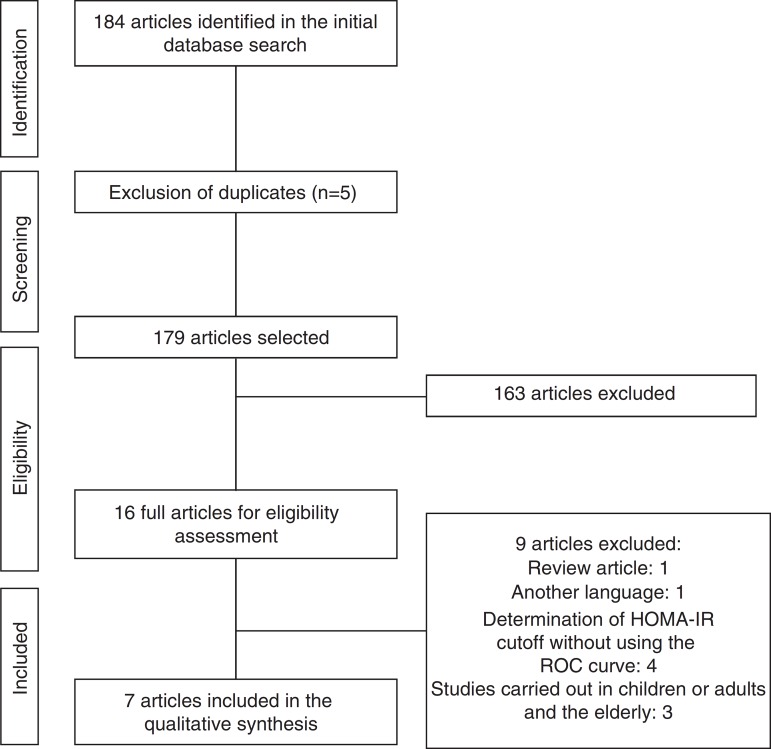
Flowchart of the process of identification and selection of articles included in the systematic review of HOMA-IR index cutoff points in adolescents.

Initially, a total of 184 articles were identified. After analyzing the titles and abstracts, we selected 16 that apparently met the inclusion criteria. After reading the full articles, nine were excluded, as they did not meet the eligibility criteria, totaling seven full articles included in the review. The flow chart of article identification and selection process is shown in Fig. 1.

The data concerning the main characteristics of the studies included in the systematic review are shown in Table 1. The studies were arranged in descending order of the obtained score, according to Strobe criteria. The median score of article quality was 14.3 (interquartile range: 12.7-17.5) points, and among the studies included, six^17^^-^ 22 obtained quality score percentage >50%.

**Table 1:** Characteristics, score and quality percentage of articles selected for inclusion in the systematic review.

Study author, year and location	Sample size	Study type	Score^a^	Percentage (%)
Yin J et al., 2013, Beijing/China	3203	Cross-sectional, cohort nested	18.5	84.0
Burrows R et al., 2015, Santiago/Chile	667	Cross-sectional, cohort nested	17.5	79.5
Kurtoglu S et al., 2010, Kayseri/Turkey	268	Cross-sectional in Pediatrics area	16.1	73.2
Singh Y et al., 2013, Delhi/India	691	Cross-sectional, cohort nested	14.3	65.0
Rocco ER et al., 2011, São Paulo/Brazil	319	School-based cross-sectional	14.0	63.6
Tresaco B et al., 2005, Zaragoza/Spain	140	School-based cross-sectional	12.7	57.7
Keskin M et al., 2005, Kayseri/Turkey	57	Cross-sectional in Pediatrics area	10.0	45.4

a Article quality assessment according to the criteria of Strengthening the Reporting of observational Studies in Epidemiology (Strobe).

Six studies,^18^^,^ 20 ^-^ 23 were carried out in foreign countries between 2005 and 2015 and one^19^ was performed with Brazilian adolescents in 2011. The smallest sample consisted of 57 participants^23^ and the largest had 3203.^22^ All analyzed studies had cross-sectional design, three^17^^,^ 20 ^,^ 22 of them nested in a cohort.

The characteristics of the samples included in the different studies and the cutoffs determined for the HOMA-IR index, with their respective sensitivities and specificities, are shown in Table 2.

**Table 2:** Sample characteristics and Homeostasis Model Assessment-Insulin Resistance index cutoff points established for adolescents in studies selected for inclusion in the systematic review.

Author	Sample characteristics	HOMA-IR	Sensitivity	Specificity
Yin J et al.	Sample: population with and without MS	2.3 (Total)	80.0% (Total)	66.0% (Total)
	Age range: 6-18 years (x±SD: 12.1±3.0)	2.6 (Pubertal)	78.0% (Pubertal)	67.0% (Pubertal)
	BMI (x±SD): ranging from 18.72±3.36 to 27.66±4.11			
	SMS: 66.1% pubertal			
	Prevalence of IR (HOMA-IR): 17.9% (Normal weight)/47.7% (Overweight)/63.2% (Obese)			
Burrows R et al.	Sample: healthy population	2.6	59.0%	87.0%
	Age range: 16-17 years (x±SD: 16.8±0.3)			
	BMI (x±SD): 0.65±1.2 (*z*-score)			
	Prevalence of obesity: 16.2%			
	SMS: sample at the age range indicative of pubertal/post-pubertal adolescents			
	Prevalence of IR (HOMA-IR): 16.3%			
Kurtoglu S et al.	Sample: obese population (100.0%)	3.82 (Pubertal girls)	77.1% (Pubertal girls)	71.4% (Pubertal girls)
	Age range: 5-18 years	5.22 (Pubertal boys)	56.0% (Pubertal boys)	93.3% (Pubertal boys)
	BMI (x±SD): 30.4±5.0 (Girls) and 30.9±4.9 (Boys)			
	SMS: 69.4% pubertal			
	Prevalence of IR (OGTT): 66.7% (Girls) and 61.7% (Boys)			
Singh Y et al.	Sample: healthy population	2.5	>70.0%	>60.0%
	Age range: 10-17 years			
	BMI (x±SD): 23.86±5.87 (Girls) and 22.81±5.64 (Boys)			
	Prevalence of OW/Obesity: 59.0%			
	SMS: 86.1% pubertal			
	Prevalence of IR (HOMA-IR): 19.7% (Normal weight)/51.7% (Overweight)/77.0% (Obese)			
Rocco ER et al.	Sample: healthy population	1.65 (Girls)	70.6% (Girls)	55.8% (Girls)
	Age range: 10-19 years	1.95 (Boys)	90.0% (Boys)	77.3% (Boys)
	BMI (x±SD): 22.5±5.9 (Girls) and 21.3±4.7 (Boys)			
	SMS (x±SD): 4.1±1.2 (Girls) and 3.2±1.5 (Boys)			
	Prevalence of IR (Percentiles of HOMA-IR): 24.0%			
Tresaco B et al.	Sample: population with and without MS	Close to 3.0	Ranging from 65.0% to 87.0%	Ranging from 64.0% to 91.0%
	Age range: 7-16 years			
	Prevalence of obesity: 48.0%			
	*SMS and prevalence of IR: not available			
Keskin M et al.	Sample: obese population (100.0%)	3.16	76.0%	66.0%
	Age (x±SD): 12.04±2.90			
	BMI (x±SD): 29.57±5.53			
	Prevalence of IR (OGTT): 44.0%			
	*SMS: not available			

SMS, sexual maturation stage; OW, overweight; HOMA-IR, Homeostasis Model Assessment-Insulin Resistance; IR, insulin resistance; MS, metabolic syndrome; OGTT, oral glucose tolerance test; x±SD, mean±standard deviation.

The adolescents included in the selected studies were individuals with metabolic syndrome or with normal glucose tolerance, with age ranging from 5 to 19 years.

The prevalence of IR varied from 16.3% to 77% and was mainly determined by the oral glucose tolerance test (OGTT) or the cutoff points established for the HOMA-IR index. In one study,^19^ IR frequency was evaluated by the percentile distribution of the HOMA-IR and was considered IR when greater than the 85th percentile.

Regarding the nutritional status of the studied adolescents, most studies^18^^,^ 20 ^,^ 23 consisted of a higher frequency of adolescents with overweight/obesity. Two studies^18^^,^ 23 were exclusively carried out in subjects classified with the diagnosis of obesity, according to the used anthropometric parameters.

Of the seven included studies, four^18^^-^ 20 ^,^ 22 included samples with the highest percentage of pubertal individuals. Two studies^21^^,^ 23 did not provide information regarding the assessed adolescents' sexual maturation stage and one study^17^ used a sample of adolescents representative of pubertal/post-pubertal individuals.

Regarding the cutoff points for HOMA-IR index, all selected studies established cutoffs using the Receiver Operating Characteristics Curve as a tool. Six studies^17^^,^ 18 ^,^ 20 ^,^ 23 preferred the use of the cutoff point with high sensitivity and specificity and one study^19^ prioritized the cutoff with greater sensitivity. The lower cutoff points found were 1.65 for girls and 1.95 for boys^19^ and the highest were 3.82 for girls and 5.22 for boys.^18^

To determine the cutoff, two studies^18^^,^ 22 took into account the adjustment according to the sexual maturation stage and two^18^^,^ 19 established cutoffs according to gender. One study^18^ proposed a cutoff adjusted for gender and sexual maturity. The cutoffs established for female adolescents were lower compared to those found for males. Regarding the studies^18^^,^ 22 that assessed prepubertal and pubertal individuals separately, data related to the prepubertal ones were not exposed in this systematic review.

The main limitations highlighted in the included studies were: small sample size, studies with cross-sectional design, no sample size calculation and sample representativeness, inability to extrapolate the results (external validation), nonspecific cutoff points for gender and sexual maturation stage and lack of standardization of laboratory methods for insulinemia detection (Table 3).

**Table 3:** Main methodological limitations and external validation of the studies selected for inclusion in the systematic review.

Author	Main methodological limitations	External validation
Yin J et al.	Lack of standardization of insulin detection methods, lack of comparison by euglycemic clamp and cross-sectional study.	Study carried out with Chinese adolescents, it is not possible to extrapolate the results to other ethnicities.
Burrows R et al.	Sample is not representative, cross-sectional study.	The cutoff is applicable in clinical practice.
Kurtoglu S et al.	Small sample size interfered in determining precise cutoff points, lack of comparison by euglycemic clamp, cross-sectional study.	Small sample size, it is not possible to extrapolate the results.
Singh Y et al.	Absence of longitudinal monitoring and comparison by the euglycemic clamp.	The cutoff point is applicable because it was obtained from a large cohort with a homogeneous sample of normal and obese individuals.
Rocco ER et al.	Lack of standardization of insulin detection methods, absence of comparison by euglycemic clamp and cross-sectional study.	The obtained data can be applied to detect a set of cardiometabolic changes.
Tresaco B et al.	Determination of a set of approximated cutoff points without establishing a single cutoff, no comparison by euglycemic clamp, cross-sectional study without considering SMS and gender to determine the cutoffs.	Restricted to the Pediatrics area. They should not be used with the general population in epidemiological studies
Keskin M et al.	Cross-sectional study, small sample size, absence of comparison by euglycemic clamp, without considering SMS and gender to determine the cutoffs.	No information

SMS, sexual maturation stage.

Of the assessed studies, one^20^ showed the possibility of result extrapolation (external validation) to other populations (Table 3).

## Discussion

Early identification of cardiovascular risk factors in adolescents is of great value in preventing chronic diseases in adulthood and the diagnosis of IR, because it has a central role in the genesis of metabolic disorders, constitutes an initial type of intervention.^24^^,^ 25

The gold standard for the detection of IR is the euglycemic clamp, recommended by the guidelines of the American Diabetes Association^26^; however, this method is not routinely used, as it is expensive and constitutes an invasive and complex procedure. The HOMA-IR index, first described by Matthews et al.^27^ in 1985, has the advantage of being a practical, fast, inexpensive method and one that has a high correlation with the euglycemic clamp (*r*=0.88; *p*<0.0001).

In a study carried out by Souza et al.^5^ with children and adolescents treated on an outpatient basis, the use of HOMA-IR (cutoff >2) was proposed^12^ for the early identification of the presence of IR, as this criterion has been able to detect a higher percentage of individuals with IR when compared to the OGTT (90.8% vs. 64.1%, respectively).

Some limitations regarding the use of the HOMA-IR index are worth mentioning, among them the use of parameters obtained in the fasting state; the use of cutoffs, which, even though are of high sensitivity and specificity, are not always devoid of errors and can include misdiagnosis; and the estimate of an overall insulin sensitivity, which can be different in the liver and peripheral tissues.^28^^,^ 29

Nonetheless, the HOMA-IR is well accepted by researchers and used in epidemiological studies to determine insulin resistance in adults, children and adolescents as a simplified option to the more expensive and sophisticated IR assessment methodologies.^6^^,^ 12 ^,^ 30 ^-^ 34 Several authors have proposed cutoff points to identify IR in adolescents based on the HOMA-IR index^9^^-^ 12 and the Receiver Operating Characteristics Curve is one of the statistical methods most commonly used for this purpose. This tool is often used in clinical and epidemiological studies that aim to determine cutoffs for diagnostic methods. This procedure takes into account the sensitivity and specificity of the test being assessed, which are related to the probability that the test will correctly distribute the studied population in not healthy/ill patients (positive) and healthy/not ill (negative) respectively.^29^^,^ 35 In the present review, it was observed that six^17^^,^ 18 ^,^ 20 ^-^ 23 of the included studies prefer to use the cutoff points with higher sensitivity and specificity. Only the Brazilian study^19^ assumed the cutoff with greater sensitivity.

According to Carrazzone et al.,^36^ screening tests require high sensitivity and moderate specificity. On the other hand, diagnostic tests require higher specificities. This allows only the actually ill individuals to be classified as having that condition. Based on this fact, it can be inferred that the cutoff with higher sensitivity, proposed in the study by Rocco et al.,^19^ can be indicated for early IR assessment as a screening method for adolescents with higher risk of developing cardiometabolic complications.

In studies in which the cutoffs were adjusted for gender,^18^^,^ 19 female adolescents had lower values for the HOMA-IR index cutoff, an event probably observed due to higher means of HOMA-IR index and higher frequencies of IR in females.

In fact, studies^4^^,^ 37 show that during adolescence, there is a physiological redistribution of fat from the extremities to the trunk, in females. Additionally, this increase in total body and abdominal fat, resulting from the sexual maturation phase and early menarche in girls, may be associated with significantly higher HOMA-IR index means. Therefore, the population of adolescents should be studied as a function of gender and sexual maturation stage. In the assessed studies, the analysis of sexual maturation was performed using the classification criteria proposed by Tanner,^38^ which consider individuals at ≥stage II for the sexual maturation stage as pubertal.

Of the included investigations, only the study by Singh et al.^20^ with Indian adolescents showed the possibility to extrapolate the results to other populations. However, the authors did not take into account the gender and stage of sexual maturation in their analyses. Additionally, it should be noted that in order to use the cutoff established in this study in Brazilian adolescents, one should take into account the differences in the prevalence of excess weight and obesity among Brazilian and Indian adolescents. The percentage of this nutritional diagnosis is lower among Brazilians (25.4% among Brazilian^39^ *vs.* 59.0% in Indian adolescents). However, the cutoff determined by the study has good sensitivity and specificity and the value can be useful in the early detection of IR.

In the study carried out by Burrows et al.^17^ with South American adolescents living in Chile, the cutoff determined for HOMA-IR index was close to that recommended by the previously cited study^20^ and an important association was found between HOMA-IR ≥2.6 and high cardiometabolic risk. It is suggested that the findings of Burrows et al.^17^ corroborate the external validity of the cutoff recommended by Singh et al.,^20^ considering how close the cutoff values determined in both studies were.

The I Guidelines of Prevention of Atherosclerosis in Childhood and Adolescence^40^ indicates the use of the cutoff proposed by Keskin et al.^23^ to determine IR in Brazilian adolescents. As there are no studies on the subject with representative samples of Brazilian adolescents, several studies carried out in the country use the recommendation proposed by the guideline^40^ for IR diagnosis.^4^^,^ 41 ^-^ 44 However, it is worth mentioning that after the publication of the guideline,^40^ other investigations were carried out using more controlled methodological procedures with larger sample sizes, which were more similar to the population of Brazilian adolescents and of which proposed cutoffs were more consistent with the physiology of these individuals.^17^^,^ 20

The cutoff proposed by the study of Rocco et al.^19^ is an option for the detection of IR among adolescents; however, as it was created for the analysis of a set of cardiometabolic alterations, it is recommended that the cutoff be used in clinical practice to screen at-risk adolescents. The cutoffs proposed by the studies of Yin et al.,^22^ Kurtoglu et al.^18^ and Tresaco et al.^21^ are geared toward the populations analyzed in the baseline studies and may not be consistent with the presence of IR in Brazilian adolescents.

Some limitations related to the design of the primary studies were recorded, such as the cross-sectional design, which prevents inferring cause and effect associations; the absence of the euglycemic clamp for comparison of this method with the HOMA-IR index; however, as mentioned before, the euglycemic clamp is not frequently performed in clinical and epidemiological studies considering its high cost; and the lack of standardization in laboratory methods for insulinemia detection, which makes it difficult to compare the original studies. Additionally, another limiting factor was the inclusion of children and adolescents in the design of the original assessed studies, without proper adjustment for the sexual maturation stage when determining the cutoffs for the HOMA-IR index.^21^^,^ 23

Regarding the interpretation of results in the present study, one must consider the probability that some articles were not found during the literature search, although the research strategy took into account the possibility of this bias in all stages, and the absence of quantitative analysis and the calculation of summary measures (meta-analysis), due to the heterogeneity of the assessed studies in terms of sampling, use of classification criteria and differentiated statistical analyses, as well as biological and social variations between the populations of the baseline studies.

In brief, the HOMA-IR index constitutes a reliable method for detection of IR in adolescents, as long as it uses cutoff points that are best suited to the reality of the population being assessed. It can be observed that the literature did not show any representative studies carried out in Brazil that aimed to determine cutoffs for IR detection using the HOMA-IR index in adolescents in the country. Therefore, it is necessary to carry out national studies with representative samples that can more reliably identify HOMA-IR index cutoff points for Brazilian adolescents.

It is expected that the results of this systematic review contribute to encourage the standardization of IR classification methods through the HOMA-IR index in adolescents and assist in the early detection of IR and cardiometabolic disease prevention in adulthood.
